# Medical cannabis for treatment-resistant combat PTSD

**DOI:** 10.3389/fpsyt.2022.1014630

**Published:** 2023-01-19

**Authors:** Nitsa Nacasch, Chen Avni, Paz Toren

**Affiliations:** ^1^Clalit Health Services Community Division, Ramat-Chen Brull Mental Health Center, Tel Aviv-Yafo, Israel; ^2^Sackler Faculty of Medicine, Tel Aviv University, Tel Aviv-Yafo, Israel

**Keywords:** cannabis, post-traumatic stress disorder (PTSD), treatment-resistant, combat PTSD, posttraumatic diagnostic scale (PDS), pittsburgh sleep quality index (PSQI), sleep quality

## Abstract

Targeting the endocannabinoid system may have a role in the treatment of post-traumatic stress disorder (PTSD). However, few studies have examined the effectiveness of cannabis on symptoms of PTSD, and more research is needed to ascertain cannabis’ effectiveness. In this retrospective naturalistic study, we followed 14 relatively mature (32-68 years of age), treatment-resistant, chronic combat post-traumatic patients who remained severely symptomatic despite treatment with many lines of conventional treatment prior to receiving medicinal cannabis. Our findings show that total sleep score, subjective sleep quality, and sleep duration significantly improved (*p* < 0.01). Total PTSD symptom score and its subdomains (intrusiveness, avoidance, and alertness) showed improvement (*p* < 0.05). However, there was no improvement in the frequency of nightmares (*p* = 0.27). The mean follow-up time was 1.1 ± 0.8 years (range of 0.5 to 3 years).

## 1. Introduction

Post-traumatic stress disorder (PTSD) is a mental disorder that can develop after a person is exposed to a traumatic event, such as threatened or actual death, serious injury, or sexual violence. Exposure can be direct, as a witness, as learning a traumatic event happened to a close person, or as repeated or extreme exposure to details of a traumatic event, such as during a line of work. The clinical presentation varies, and symptoms may include: Fear-based reexperiencing (such as in intrusive recollections, nightmares, or dissociative states); Intense physiological or psychological distress when exposed to triggering cues that remind of the traumatic events and persistent avoidance of such cues (internally or externally); Negative alterations in cognition or mood including difficulty in remembering important parts of the event, negative expectations about oneself, others or the future. Persistent negative mood states with decreased ability to feel positive feelings. Diminished interest in previously enjoyed activities and feelings of detachment or estrangement from others; Alterations in arousal and reactivity, including irritable behavior, hypervigilance, exaggerated startle response, and more (1). According to different guidelines for the treatment of post-traumatic stress disorder (PTSD), including the American Veterans Affairs (VA) and American Department of Defense (DoD) guidelines (2), and the International Society for Traumatic Stress Studies (ISTSS) (3), the first line treatment of PTSD includes psychotherapy and/or SSRIs or SNRIs. The most evidence-based and effective treatments are cognitive behavioral therapy (CBT), specifically prolonged exposure (PE), as well as eye-movement desensitization and reprocessing (EMDR) (4, 5). However, a large proportion of patients avoid psychological treatment, and the dropout rate among veterans is high (6). Remission rates with medication are only around 20 to 30%, and their side effects cause low compliance resulting in poor efficacy (7, 8). Considering the limitations of these treatments and the need for effective treatment for patients diagnosed with PTSD, an interest in medical cannabis for PTSD has risen in recent years.

In the past two decades, there has been growing literature implicating the involvement of the endocannabinoid system (eCS) in the etiology of PTSD (9, 10). The endocannabinoid system is a system of cannabinoids produced in our body and includes endogenous cannabinoids, such as N-arachidonoyl ethanolamine (anandamide) and 2-arachidonoyl glycerol (2-AG) and CB_1_ and CB_2_ receptors. CB_1_ receptors are located primarily in the brain and are widely located in the same areas in the brain that are involved in PTSD - the amygdala, hippocampus, and prefrontal cortex (11). CB_2_ receptors are located primarily in peripheral immunological tissue, although their presence in the central nervous system has also recently been documented. In fact, activating circuits and mechanisms involving CB receptors are similar to pathways involved in PTSD (12).

By activating CB_1_ receptors in the amygdala, cannabis can potentially reduce fear, anxiety, and aversive memories (13–18). By stimulating CB_1_ receptors in the prefrontal cortex, cannabis may increase serotonin levels, thus reducing depression, and improving mood, memory, and neurogenesis (17). Cannabis may also decrease hyperarousal and intrusive memories by activating CB_1_ receptors in the hippocampus and thus might help to reduce PTSD symptoms (13). Furthermore, studies demonstrate a lower concentration of endocannabinoids in patients with PTSD. For example, in a study by Hill et al., endocannabinoid levels were measured in 46 people (24 with PTSD and 22 without PTSD) who were exposed to the 9/11 terror attack. They found that 2-arachidonoylglycerol (2-AG) levels were significantly lower among those who developed PTSD (19).

Cannabinoids are a group of active compounds found in the cannabis plants. The most well-known cannabinoids are Δ-9-tetrahydrocannabinol (THC) and cannabidiol (CBD), which bind to the endocannabinoid receptors mentioned above. Both THC and CBD act on cannabinoid receptors CB_1_.CBD acts on CB_2_ too as well as other targets such as Fatty acid amide hydrolase (FAAH), serotonin 5-HT_1A_ receptor and more (20). THC is the primary psychoactive ingredient in cannabis. It reduces anxiety (but can also increase anxiety), improves sleep, reduces nightmares, increases hunger, and helps in the extinction of fear memory (15, 21, 22). Cannabidiol (CBD) is a non-psychotomimetic cannabinoid that causes the least side effects while reducing the anxiety and psychoactive symptoms caused by THC (23). CBD significantly reduces the consolidation of aversive memories and has an anti-inflammatory effect with neuroprotective, analgesic, sedative, antiemetic, antispasmodic, anti-inflammatory, and anxiolytic properties (24). Cannabis use is not without its dangers. Cannabis use disorder afflicts about 22% of cannabis users, and 13% develop cannabis dependence (25). Cannabis use may increase the risk of psychosis and hinder its treatment (26, 27).

Literature reviews from the past two years that have examined the effectiveness of cannabis on PTSD symptoms count a small number of heterogeneous studies (open-label, longitudinal, and retrospective studies) with methodological problems and many limitations (28, 29). To date, there were only two randomized, controlled clinical trials for PTSD patients. The first used the synthetic cannabinoid nabilone (30), and the second used smoked cannabis but for only three weeks and was underpowered to detect significant differentiation from placebo (31). The conclusions of the few systematic reviews examining the effectiveness of cannabis on PTSD symptoms are that cannabis and synthetic cannabinoids may have a role in the treatment of PTSD, but there is currently limited evidence regarding their safety and efficacy (28, 29). Therefore, additional research is needed to better understand the effectiveness and safety of cannabis in the treatment of PTSD.

Israel has one of the longest-running medical cannabis programs in the world, starting in the 1990s (32). However, the use of medical cannabis for PTSD in Israel began only in 2014 (33). The use of cannabis for PTSD increased to almost 10% of total licenses by 2018, although Israel has a lower prevalence of PTSD than the USA (1.5% vs. 6.8%, respectively) (32). Medical cannabis is available as dried buds for inhalation or smoking and as an oil for sublingual ingestion. License for the medicinal use of cannabis for PTSD requires an application sent to the Medical Cannabis Unit at the Ministry of Health on behalf of a patient by the treating psychiatrist. Licenses are valid for a year and require that the psychiatrist request extending the permit annually (34). The initial dose is 20 grams per month, with possible increments of 10 grams at the treating physician’s request. We advised patients to start with a low-THC concentration strain. The cannabis is dispensed at specialized pharmacies given special permit by the Ministry of Health. According to the Ministry of Health guidelines at the time, medical cannabis may be prescribed to patients who are diagnosed with moderate PTSD and above, lasting at least three years, and characterized by great distress. Cannabis will only be given after at least two trials with different drugs and two psychological interventions have been attempted. Contraindications to treatment include a history of psychosis or drug abuse (33). In this study, we examined the effectiveness of medical cannabis among patients suffering from chronic combat-PTSD in a clinical setting in Israel.

## 2. Materials and methods

The study is a retrospective naturalistic study that used data meticulously gathered in a real-life clinical setting during routine follow-up unrelated to the study. It was approved by the institutional review boards and was conducted following the International Conference on Harmonization guidelines and ethical principles of the Declaration of Helsinki (approval# 0118-19-COM1). Patients consented verbally for their anonymized data to be examined with no patient declining.

Since 2015, our specialized psychiatric trauma unit has offered medical cannabis to treatment-resistant combat-PTSD veterans who fit the MOH criteria. Patients complete questionnaires every six months as part of their treatment follow-up and before applying for a medical cannabis license. The two questionnaires used are the Hebrew versions of the PSQI (35) and PDS (36).

The Pittsburgh Sleep Quality Index Hebrew version (PSQI-H) is a self-administered ten-question questionnaire (Cronbach’s alpha = 0.72). A previous study had the original English questionnaire translated, back-translated, refined, and later validated by fluent speakers of both English and Hebrew. We used the following questions: (A) subjective quality of sleep rated between 0 (very good) to 3 (very bad), (B) duration of sleep in hours, (C) Inability to fall asleep within 30 minutes rated between 0 (not in the past month) to 3 (three or more times a week), and (D) total score calculated from all ten questions (higher scores indicate worse sleep quality). The Posttraumatic Diagnostic Scale (PDS) is a 49-item measure that assesses all the DSM-IV criteria for PTSD and measures symptom severity. For this study, we only used part three of the questionnaire, which includes 17 questions that measure the severity/frequency of PTSD symptoms in the past month, as the diagnosis was already well established. Questions range between 0 (never or once in the past two weeks) to 3 (at least five times a week). Symptoms can be separated into reexperiencing, avoidance, and arousal clusters. Although a formal validation of the Hebrew translation of the PDS has not been published, several theses have implemented similar translations. Weisblum (37) reported that her translation had a satisfactory internal consistency level (Cronbach’s Alpha = 0.90) in a study of parents whose children underwent heart catheterization or cardiac surgery. We used Weisblum’s translation as we found it most accurate and faithful to the original English questionnaire.

We extracted data from the questionnaires given to patients diagnosed with combat-PTSD who started treatment with cannabis at the trauma unit at the Brüll Mental Health Center, Tel Aviv, between 2015 and 2018. The questionnaires were completed as part of the patients’ standard assessment just before receiving medical cannabis and every six months afterward.

This study included all patients who were treated with cannabis for combat-PTSD and who completed PDS and PSQI questionnaires both before starting treatment with cannabis and at least once afterward (no less than six months after starting cannabis treatment). In addition, these patients had to fit the criteria for medical cannabis treatment laid out by the Ministry of Health: (1) PTSD lasting at least three years (2) Moderate or severe severity of PTSD (3) At least two previous medications were used for at least two months, including SSRIs or SNRIs (4) Two psychotherapeutic treatments. Excluded for treatment by the MOH criteria: any history of psychosis or current psychosis or any substance abuse (specifically, patients in our sample stated they had no previous cannabis use).

### 2.1. Statistical analysis

We compared each patient’s post- and pre-treatment questionnaires using SPSS with paired samples t-test for ratio and quasi-interval variables, Wilcoxon signed-rank test for ordinal variables, and Hodges-Lehmann estimator for confidence intervals for ordinal scale variables. We used the latest questionnaire when more than one post-treatment questionnaire was available. In addition, we used Mann-Whitney U Test to compare gender-specific differences in total PSQI and PDS symptom severity scores.

## 3. Results

After screening all patients’ files between 2015-2018, we extracted data from the files of 14 men and women (12 men, 2 women) who met the study’s inclusion and exclusion criteria. No patient files were excluded. Patient’ mean age was 49.5 ± 13.1. Most patients (86%) were currently married and had children. All cases were chronic, with average years since trauma being 27.6 ± 12.3 (range 10-45) and years under treatment prior to being prescribed cannabis averaged 7.7 ± 5.2 (range 1-17). The mean follow-up was 1.1 ± 0.8 years (0.5-3). Comorbidities and previous treatments appear in Table 1.

**TABLE 1:** Patients’ demographic data.

Patient characteristics	*N* = 14
Male, *n* (%)	12 (86)
Age, mean ± SD (range)	49.5 ± 13.1 (32-68)
Married, *n* (%)	12 (86)
Has children, *n* (%)	12 (86)
Physical injury during the traumatic event, *n* (%)*	5 (36)
Years since trauma, mean ± SD (range)	27.6 ± 12.3 (10-45)
Successfully completed prolonged exposure therapy (PE), *n* (%)	10 (71)
Years in treatment as usual when cannabis initiated, mean ± SD (range)	7.7 ± 5.2 (1-17)
Follow-up time in years following cannabis initiation, mean ± SD (range)	1.1 ± 0.8 years (0.5-3)
Comorbid depression, *n* (%)	10 (71)
Comorbid obsessive-compulsive disorder, *n* (%)	5 (36)
Attempted treatment with at least one SSRI, *n* (%)	14 (100)
Attempted treatment with at least one SNRI, *n* (%)	6 (43)
Attempted treatment with bupropion, *n* (%)	3 (21)
Attempted treatment with anti-psychotics, *n* (%)	1 (7)
Treated with medication for sleep disturbances, *n* (%)	12 (86)

Characteristics of study participants (*N* = 14): Demographic data, trauma details, cannabis initiation, follow-up, comorbidities, and previous treatments. *None of the patients suffered head trauma.

**FIGURE 1:**
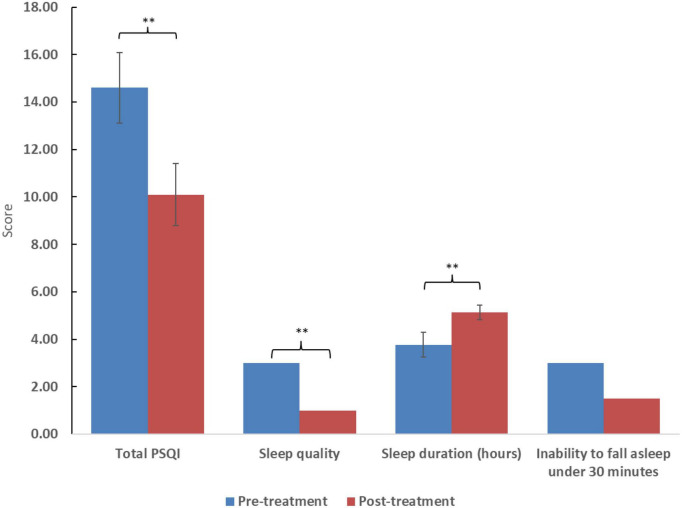
Scores of the posttraumatic diagnostic scale (PDS) and selected subdomains. Higher scores reflect worse symptom severity. Significance: *p < 0.05, ^**^p < 0.01. For ratio and quasi-interval scales, means and standard errors are displayed, while medians are displayed for ordinal scales.

**FIGURE 2:**
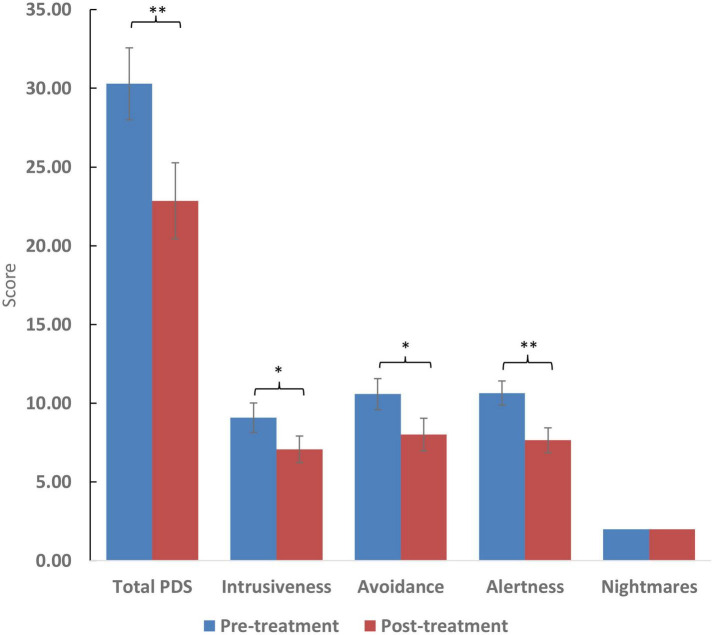
Scores of the pittsburgh sleep quality index (PSQI) and selected questions. Higher scores reflect worse symptom severity except in the case of sleep duration which is measured in hours. Significance: ^**^p < 0.01. For ratio and quasi-interval scales, means and standard errors are displayed, while medians are displayed for ordinal scales.

After treatment with cannabis, total sleep score, subjective sleep quality, and sleep duration significantly improved (*p* < 0.01). However, improvement in difficulty falling asleep in under 30 minutes was statistically only marginally improved (*p* = 0.58). Total PTSD symptom score and its subdomains (intrusiveness, avoidance, and alertness) showed improvement (*p* < 0.05). However, there was no improvement in the frequency of nightmares (*p* = 0.27). The two women included displayed similar improvement to the 12 men included. Clinical results appear in Table 2 and Figures 1, 2.

**TABLE 2:** Cannabis treatment results by clinical scales.

	n	Before treatment: mean ± SD / median (IQR)	After treatment: mean ± SD / median (IQR)	Test statistic	Effect size	Difference	95% Confidence interval		*P*
**Pittsburgh sleep quality index (PSQI)**									
Total ^t^	10	14.6 ± 4.1	10.1 ± 4.7	*t* = −4.4	−1.40	−4.5	−6.8	−2.2	<0.01**
Sleep quality ^w^	14	3.0 (1.0)	1.0 (1.25)	*z* = −3.1	−0.59	−1.5	−2.0	−1.0	<0.01**
Sleep duration (hours) ^t^	11	4.3 ± 2.4	5.2 ± 1	*t* = 3.9	1.17	0.9	0.6	2.1	<0.01**
Inability to fall asleep under 30 min ^w^	14	3.0 (1.5)	1.5 (3.0)	*z* = −1.9	−0.36	−0.5	−1.5	0.0	0.058
**Posttraumatic diagnostic scale (PDS)**									
Total symptom severity^t^	14	30.3 ± 8.5	22.9 ± 9.0	*t* = −3.3	−0.86	−7.4	−12.3	−2.5	<0.01**
Intrusiveness ^t^	14	9.1 ± 3.5	7.1 ± 3.2	*t* = −2.4	−0.65	−2.0	−3.8	−0.2	<0.05*
Avoidance ^t^	14	10.6 ± 3.7	8.0 ± 3.9	*t* = −2.5	−0.66	−2.6	−4.8	−0.3	<0.05*
Alertness ^t^	14	10.7 ± 2.9	7.6 ± 3.0	*t* = −3.1	−0.83	−3.1	−5.1	−0.9	<0.01**
Nightmares ^w^	14	2.0 (2.0)	2.0 (1.0)	*z* = −1.1	−0.21	−0.5	−1.0	0.5	0.27

Participants’ PSQI and PDS symptom severity test scores before and after treatment with cannabis. Lower scores imply improvement (excluding sleep duration, measured in hours). Different sample size between tests is due to the incomplete filling of questionnaires by some of the patients. The statistical tests used were a *t*-test for ratio and quasi-interval variables, Wilcoxon signed-rank test for ordinal variables, and a Hodges-Lehmann estimator for confidence intervals for ordinal scales variables. Each variable is labeled with the statistical method used according to the following: w = Wilcoxon signed-rank test; difference and confidence intervals were calculated using related-samples Hodges-Lehmann median difference; t = paired samples *t*-test; difference denotes the difference in means. *p < 0.05 and **p < 0.01.

Regarding gender, we found post-treatment PDS scores to be statistically significantly lower in women compared to men (7 vs. 23.7, *p* < 0.05) without significant difference pre-treatment (23.5 vs. 30, *p* = 0.44). In addition, PSQI total scores post-treatment were marginally statistically lower in women (3 vs. 9.9. *p* = 0.051), but pre-treatment was unavailable as one of the women did not complete the entire PSQI questionnaire correctly.

All patients reported only using cannabis before going to sleep, and no patient reported or displayed any sign of cannabis misuse, abuse, or side effects in repeated psychiatric interviews during follow-ups. No patient stopped using cannabis during the follow-up period. The amount of cannabis did not exceed 20 grams per month, but the minimal amount used could not be discerned.

## 4. Discussion

Many studies have found a possible role for medical cannabis in the treatment of PTSD with no definite conclusion as of yet due to the paucity of studies and methodological limitations (38). We aimed to add to the building body of knowledge by demonstrating the possible usefulness of cannabis for at least a subset of patients with specific properties. In this retrospective naturalistic study, we used the files of patients diagnosed with chronic combat-PTSD who were treated with cannabis after exhausting all other treatment options. Our study is unique primarily due to the nature of the participants. All participants were relatively mature (mean age was 50 years old), treatment-resistant, chronic combat-PTSD patients who had undergone several pharmacological treatment lines and prolonged exposure therapy prior to receiving treatment with cannabis, and were treated in the same clinic by the same expert psychiatrist in the field of PTSD (NN). Prior to treatment with cannabis, patients continued to suffer from moderate to high-level PTSD symptoms despite being in treatment for an average of 7 years. Cannabis was only prescribed after receiving the standard treatment and not as primary treatment, with none of the patients having a background of substance abuse, and all of them had never used cannabis before the study. In addition, the study’s uniqueness is also in the long follow-up of patients, averaging over a year (range 0.5-3 years), enabling the assessment of the effect of cannabis on PTSD symptoms, possible side effects, and the possibility of addiction.

The study’s findings show an overall improvement in sleep quality and duration, as well as a decrease in PTSD symptoms. According to the PDS questionnaire, there was a reduction of at least 20% in PTSD symptoms in over 65% of patients, with nearly 80% showing improvement. Surprisingly, unlike other studies (28), the decrease in nightmares was observed but was not significant, maybe due to the small number of participants.

The widespread use of cannabis today in patients with PTSD, whether legally or otherwise, is often problematic due to the onset of use at a young age and the potential for addiction and dependence (39). There is also a problem with patients preferring cannabis treatment as their first treatment over evidence-based pharmacological and psychological treatments (40). In our study, the pre-selection process created a mostly homogeneous group of relatively mature patients without prior use of cannabis who were treatment-resistant to several evidence-based therapies. By selecting this group, we have shown that cannabis may be beneficial and alleviate some suffering among patients resistant to evidence-based treatments.

It is known that subjects suffering from PTSD tend to use cannabis as a form of self-medication more widely than the general population (41, 42), thus suffering from high rates of substance-related disorders. Our long-term follow-up allowed us to see that none of the patients suffered from side effects and symptoms of addiction. It is possible that the characteristics of our participants (mature, in treatment for many years by the same physician, mostly married with children) led to a more responsible use (just before bedtime, in a fixed amount, without recreational use) which played a role in the good outcome.

The limitations of the study should be noted. First, the study is retrospective, and does not include placebo or control groups. However, it may be argued that as patients were stable in their symptoms prior to cannabis treatment, they were their own controls. Second, while we ensured that the dose of cannabis did not exceed 20 grams per month, the type of compounds, the ratio of CBD to THC, the route of administration, timing of usage, and the actual amount used were not controlled as patients are mainly free to try different strains without notifying their psychiatrist. Third, formal validation of the Hebrew translation of the PDS has not been published as of yet, though several translations were used in various theses and studies. Therefore, we used an unpublished translation that we found most accurate and faithful to the original. Lastly, the sample is relatively small, consisting of only 14 patients, and the majority (86%) were men. Although it seemed that women benefited more from cannabis treatment, the sample size was too small (a total of two women) to draw any conclusions. The gender difference may warrant further study.

To the best of our knowledge, this is the first published study examining long-term cannabis efficacy in chronic combat treatment-resistant PTSD patients. The study we conducted is consistent with existing literature which indicates a decrease in PTSD symptoms under medical cannabis treatment. There are still very few studies examining the effectiveness of medical cannabis for PTSD patients. One needs to remember that the endocannabinoid system works in different ways, even in the same disorder and among different genders and ages, which may also affect outcomes. Our results suggest the importance of the selection process in terms of patients receiving medical cannabis. Future research should clarify the long-term effects of cannabis on different groups of patients suffering from PTSD.

## Data availability statement

The raw data supporting the conclusions of this article will be made available by the authors, without undue reservation.

## Ethics statement

The study involving human participants was reviewed and approved by the Institutional Review Board of Clalit Health Services and was conducted following the International Conference on Harmonization guidelines and ethical principles of the Declaration of Helsinki (approval# 0118-19-COM1). Written informed consent for participation was not required for this study in accordance with the national legislation and the institutional requirements.

## Author contributions

NN: study design, data collection, and writing. CA: data collection, statistical analysis and interpretation, and writing and editing. PT: supervision and writing review and editing. All authors contributed to the article and approved the submitted version.
